# Four Cases of Chylous Ascites following Robotic Gynecologic Oncological Surgery

**DOI:** 10.1155/2014/953965

**Published:** 2014-03-04

**Authors:** Ahmet Göçmen, Muhittin Eftal Avcı, Fatih Şanlıkan, Mustafa Gazi Uçar

**Affiliations:** ^1^Obstetrics and Gynecology Department, Umraniye Education and Research Hospital, 34766 İstanbul, Turkey; ^2^Departments of Aegean Obstetrics and Gynecology, Training and Research Hospital, 35170 Izmir, Turkey; ^3^Departments of Obstetrics and Gynecology, Konya Education and Research Hospital, 423030 Konya, Turkey

## Abstract

Chylous ascites is an uncommon form of ascites characterized by milky-appearing fluid caused by blocked or disrupted lymph flow through chyle-transporting vessels. The most common causes of chylous ascites are therapeutic interventions and trauma. In this report, we present four cases of chylous ascites following robot-assisted surgery for endometrial staging and the treatment strategies that we used. After retroperitoneal lymph node dissection, leaving a drain is very useful in diagnosing chylous ascites and observing its resolution; furthermore, the use of octreotide in conjunction with TPN appears to be an efficient treatment modality for chylous ascites and should be considered before any invasive intervention.

## 1. Introduction

Chylous ascites is an uncommon form of ascites characterized by milky-appearing fluid caused by blocked or disrupted lymph flow through chyle-transporting vessels. The most common causes of chylous ascites are therapeutic interventions and trauma. The increase in the incidence of chylous ascites is attributed to the longer survival of patients with cancer and more aggressive abdominal, retroperitoneal, and cardiothoracic interventions, despite the lack of recent large studies [1]. The recommended treatment options for chylous ascites are therapeutic paracentesis, dietary control with a high-protein, low-fat, medium-chain triglyceride- (MCT-) based diet, total parenteral nutrition (TPN), somatostatin, and surgical intervention [2].

In this study, we present four cases of chylous ascites following robot-assisted surgery for gynecologic malignancies and their management.

## 2. Case Presentations

**Case 1**

A 58-year-old woman, gravida 6, para 6, with a two-month history of vaginal bleeding was referred to our hospital. Transvaginal ultrasonography revealed a solid 12 × 17 mm lesion in the endometrial cavity. The patient was diagnosed with a carcinosarcoma with heterologous elements following an endometrial biopsy. The patient underwent robot-assisted endometrial staging via the da Vinci S surgical system (Intuitive Surgical Sunnyvale, CA). The procedure included hysterectomy, bilateral salpingo-oophorectomy (BSO), and pelvic and paraaortic lymph node dissection (PPLN) up to renal vein. The patient was discharged on the third postoperative day without any complication. The final pathological finding was a stage IB grade III carcinosarcoma with heterologous elements. A total of 35 removed lymph nodes were free of disease. The patient was readmitted to the hospital two weeks after surgery due to abdominal distension, nausea, dyspnea, and labial swelling. Diagnostic paracentesis was performed, and 2.5 liters of milky fluid was withdrawn. Laboratory analysis confirmed chylous ascites. The patient's oral intake was restricted, and a high-protein, low-fat diet with medium-chain triglyceride supplementation was started. One week later, a second paracentesis to relieve the abdominal distention was performed. Octreotide, a somatostatin analog, was administered subcutaneously at a dose of 100 mcg three times per day after the ascites persisted despite dietary management. Octreotide was maintained with TPN for seven days. There was a good response to this treatment, and after the resolution of the chylous ascites, the patient completed platin-based chemotherapy and radiation therapy. Over a 17-month follow-up period, there was no evidence of chylous ascites.

**Case 2**

A 50-year-old woman, gravida 4, para 2, was admitted to our clinic for routine postmenopausal assessment. An endometrial thickness of 12 mm was measured on transvaginal ultrasonography. Endometrial sampling revealed a grade II endometrioid type adenocarcinoma. The patient underwent endometrial staging with the da Vinci surgical system, which included hysterectomy, BSO, and PPLND. The final pathological finding was a stage IB endometrial adenocarcinoma. A total 47 removed lymph nodes were free of disease. On the third postoperative day, approximately 1.3 liters of milky fluid accumulated in a drain container. Suspicion of chylous ascites was confirmed by laboratory analysis. Dietary management with a high-protein, low-fat diet with medium-chain triglyceride supplementation was started. The patient's oral intake was restricted, and bowel rest and TPN were initiated. No response was observed over two days, and subcutaneous 100 mcg 3 × 1/day octreotide was then started. The ascites resolved in 11 days, and the drain was removed. The patient remained asymptomatic with no evidence of chylous ascites at follow-up after seven months.

**Case 3**

A 54-year-old nulligravida woman presented with postmenopausal bleeding. Leiomyosarcoma was diagnosed after endometrial sampling. Robot-assisted endometrial staging was performed. The procedure included hysterectomy, BSO, and PPLND up to the left renal vein. On the second postoperative day, the amount of fluid increased, and the serous content of the fluid became a milky, whitish fluid. Laboratory analysis of the fluid revealed chylous ascites. Dietary management was started with oral intake restriction and a high-protein diet. Resolution was achieved with conservative management; no other interventions, including TPN or somatostatin, were used. The patient was discharged four days after surgery. After conservative management, no recurrence was observed over nine months of follow-up.

**Case 4**

A 56-year-old woman, gravida 3, para 3, was admitted to our clinic for postmenopausal bleeding. The result of an endometrial biopsy was grade 2 endometrioid adenocarcinoma. Robot-assisted laparoscopic surgery involving hysterectomy, BSO, and PPLN up to the level of left renal vein was performed with the da Vinci S surgical system (Intuitive Surgical Sunnyvale, CA). The final pathological finding was a stage IB endometrial adenocarcinoma. On the second postoperative day, approximately 0.5 liters of milky fluid accumulated in a drain container. Laboratory examination confirmed the diagnosis of chylous ascites. Dietary management with a high-protein, low-fat diet and medium-chain triglyceride supplementation was started. The patient's oral intake was restricted, and bowel rest and TPN as well as 100 mcg 3 × 1/day octreotide were initiated subcutaneously. The ascites resolved in five days, and the drain was removed. The patient remained asymptomatic with no evidence of chylous ascites at follow-up after 3 months.

## 3. Discussion

There are several causes of chylous ascites, including abdominal malignancy, cirrhosis, infectious etiologies, trauma, inflammatory reasons, abdominal surgery, and miscellaneous disorders. Surgical lymph node dissection is the most common iatrogenic cause, and a small amount of lymphatic leakage may occur after every lymphadenectomy, but such leaks generally seal spontaneously without development of symptomatic ascites, whereas damage to a major lymphatic channel during surgery may cause the continuous collection of lymphatic fluid in the retroperitoneal and abdominal cavities [3].

After abdominal surgery, chylous ascites can occur early (after approximately one week) due to the disruption of the lymphatic vessels or late (several weeks to months) due to adhesions or the extrinsic compression of lymphatic vessels [4]. According to this definition, three of our four cases occurred in the early period (two of them occurred on the second postoperative day, and one occurred on the third postoperative day) and one case occurred in the late period (two weeks after operation).

The most important diagnostic tool in evaluating and managing patients with ascites is abdominal paracentesis. The triglyceride levels in ascitic fluid are critical in diagnosing chylous ascites [5]. Although some authors use a cut-off value of 110 mg/dL, triglyceride values are typically above 200 mg/dL, as in all of our cases.

In the management of postoperative chylous ascites, the first step should be reducing lymph flow through the obstructed or disrupted lymph vessels. Due to limited data, a feasible initial modality is a high-protein and low-fat diet with medium-chain triglyceride (MCT) supplementation. MCTs are absorbed directly into intestinal cells and transported as free fatty acids and glycerol directly to the liver via the portal vein and bypassing the lymphatics. Hence, a low-fat diet with MCT supplementation reduces the production and flow of chyle [5, 6]. Only this treatment has been sufficient in treating chylous ascites in one of our patients.

We used TPN and octreotide (a long-acting synthetic analog of somatostatin) in three of our four cases. TPN provided nutritional, caloric energy support and the requisite conditions for tissue repair and lymphatic healing and reduced lymph production and flow by enabling the bowel to rest [7].

The use of octreotide in cases of chylous ascites is a recent modality that has attracted much attention over the past decade. Somatostatin is known to inhibit a variety of gastrointestinal functions and to exert neural control over many physiological processes. The clinical efficiency of somatostatin is hampered by its short half-life in the circulation (less than three minutes). Hence, octreotide acetate, a synthetic peptide that possesses the biological activity of somatostatin but remains active for more than 90 minutes, was developed. Octreotide is much more stable in the circulation and is more potent with respect to many inhibitory functions than native somatostatin. The development of synthetic analogs has led to the treatment of clinical disorders such as acromegaly, hormone-secreting tumors of the gastrointestinal tract, and portal hypertensive bleeding. Given this expansive range of effects, it is not surprising that somatostatin has been the subject of intensive investigation [8].

In the treatment of chylous ascites, the proposed mechanism through which octreotide stops chylous effusion is by diminishing the intestinal absorption of fats, diminishing the triglyceride concentration in the thoracic duct, and decreasing lymph flow in the major lymphatic channels [9].

Thus, we have added octreotide to the therapeutic regimen for our patients and have obtained successful outcomes for treating chylous ascites, as demonstrated in a number of case reports, although there is not yet enough data in the literature due to the rarity of chylous ascites.

In summary, in gynecologic oncologic surgical procedures, postoperative chylous ascites is an infrequent yet significant complication that may occur despite the use of meticulous and minimally invasive dissection techniques and new technologies such as the da Vinci robot, which provides enhanced intraoperative visualization and convenience for radical surgery. After retroperitoneal lymph node dissection, leaving a drain is very useful in diagnosing chylous ascites and observing its resolution; furthermore, the use of octreotide in conjunction with TPN appears to be an efficient treatment modality for chylous ascites and should be considered before any invasive intervention.
